# Chitosan-Covered Calcium Phosphate Particles Co-Loaded with Superoxide Dismutase 1 and ACE Inhibitor: Development, Characterization and Effect on Intraocular Pressure

**DOI:** 10.3390/pharmaceutics15020550

**Published:** 2023-02-07

**Authors:** Ekaterina Popova, Olesya Matveeva, Olga Beznos, Victoria Tikhomirova, Elena Kudryashova, Yuri Grigoriev, Natalia Chesnokova, Olga Kost

**Affiliations:** 1 Chemistry Faculty, M.V. Lomonosov Moscow State University, 119991 Moscow, Russia; 2 Helmholtz National Medical Research Center of Eye Diseases, 105062 Moscow, Russia; 3 Shubnikov Institute of Crystallography, Federal Scientific Research Center Crystallography and Photonics, Russian Academy of Sciences, 119333 Moscow, Russia

**Keywords:** calcium phosphate nanoparticles, chitosan, superoxide dismutase 1, angiotensin-converting enzyme inhibitor, intraocular pressure, eye diseases, glaucoma, drug delivery

## Abstract

Improvement of the efficiency of drug penetration into the eye tissues is still an actual problem in ophthalmology. One of the most promising solutions is drug encapsulation in carriers capable of overcoming the cornea/sclera tissue barrier. Formulations on the base of antioxidant enzyme, superoxide dismutase 1 (SOD1), and an inhibitor of angiotensin-converting enzyme, enalaprilat, were prepared by simultaneous inclusion of both drugs into calcium phosphate (CaP) particles in situ with subsequent covering of the particles with 5 kDa chitosan. The formulations obtained were characterized by dynamic light scattering and scanning electron microscopy. Hybrid CaP-chitosan particles co-loaded with SOD1 and enalaprilat had a mean hydrodynamic diameter of 120–160 nm and ζ-potential +20 ± 1 mV. The percentage of the inclusion of SOD1 and enalaprilat in hybrid particles was 30% and 56%, respectively. The ability of SOD1 and enalaprilat to reduce intraocular pressure (IOP) was examined in vivo in normotensive Chinchilla rabbits. It was shown that topical instillations of SOD1/enalaprilat co-loaded hybrid particles were much more effective in decreasing IOP compared to free enzyme or free enalaprilat and even to the same particles that contained a single drug. Thus, the proposed formulations demonstrate potential as prospective therapeutic agents for the treatment of glaucoma.

## 1. Introduction

Instillation of eye drops into the lower conjunctival sac is the simplest and most patient-friendly approach to ophthalmic therapy. However, conventional formulations often exhibit fast elimination, poor bioavailability, insufficient contact with epithelium, and difficulties penetrating ocular tissue barriers [1]. Instilled drugs in the form of aqueous solution are almost instantly eliminated via the nasolacrimal drainage system and blinking; therefore, some eye diseases, including glaucoma, require frequent instillations of the drug throughout the day. To improve the retention time and availability of the drugs without the development of local and general side effects, various delivery systems have been suggested [1,2,3,4].

One of the most promising ways to enhance the effectiveness of the drug is its encapsulation into vehicles of various natures capable of penetrating the corneal/sclera tissue barrier (for reviews, see [1,2,5,6,7,8]). The encapsulation of drugs into nanosized calcium phosphate (CaP) vehicles seems to be very positive for the use in ophthalmology since CaP-nanoparticles are biocompatible, biodegradable, and non-immunogenic [9,10,11]. CaP-nanoparticles themselves have no inherent toxicity but can lead to an increase in the intracellular calcium concentration after endosomal uptake and lysosomal degradation. CaP-nanoparticles absorbed by cells dissolve with the formation of non-toxic calcium and phosphate ions, which are removed from the cytoplasm in a few hours [12]. The synthesis of CaP-nanoparticles is rather simple and inexpensive [13], and these particles are capable of incorporating both high and low-molecular-weight compounds [14,15,16,17]. We have previously demonstrated that the inclusion of low molecular weight drugs, β-adrenoblocker timolol [16], and the inhibitors of angiotensin-converting enzyme (ACE), into CaP-nanoparticles increased the effects of these drugs on the intraocular pressure (IOP) [17,18,19], while the inclusion of the antioxidant enzyme, superoxide dismutase 1 (SOD1), into such particles was shown to be effective in the treatment of oxidative stress and inflammation in immunogenic uveitis [20].

However, CaP-particles possess a negative surface charge [16,17], which might impair drug penetration into the eye due to the negatively charged mucin layer on the anterior eye surface [21,22]. Thus, the coating of CaP-particles with common positively charged mucoadhesive biopolymer chitosan [23,24] can enhance the affinity for the eye surface and increase the bioavailability of the included drug [18,19] without toxicity risks [25]. We have shown previously that hybrid chitosan-covered CaP-particles containing the ACE inhibitor, enalaprilat, demonstrated elevated retention time in the tear fluid and enhanced efficacy in lowering IOP with prolonged effect compared to both enalaprilat in solution and enalaprilat within uncovered CaP-particles [19].

Here, we develop hybrid particles on the base of inorganic CaP-core covered with 5 kDa chitosan and incorporating two different agents simultaneously—ACE inhibitor, enalaprilat, and the enzyme, SOD1. ACE inhibitors are known to reduce IOP and ischemia of the eye [26,27,28,29,30,31] via reducing the concentration of the ACE product, angiotensin II [32,33,34]. SOD1 controls the concentration of the superoxide anion, preventing it from interacting with nitric oxide. Thus, SOD1 does not allow the concentration of nitric oxide to fall. A decrease in the nitric oxide level impairs the outflow of intraocular fluid and increases ocular hypertension [35]. In addition, the effectiveness of the topical application of SOD1 was demonstrated for the treatment of oxidative stress and inflammatory processes in the eye [36,37], preventing the apoptosis of retina nerve cells, which is the cause of vision loss in neurodegenerative eye diseases including glaucoma [38]. Thus, these different agents are capable of reducing IOP and, at the same time, have anti-ischemic and antioxidant actions. The aim of this study was to find out whether the inclusion of two different agents in hybrid chitosan-covered CaP nanoparticles could be beneficial for the decrease in IOP and whether the prospective formulation has promise as an anti-glaucoma remedy.

## 2. Materials and Methods

### 2.1. Materials

All chemicals were of analytical grade and used without further purification. Recombinant human SOD1 was purchased from Life Science Advanced Technologies (St. Petersburg, Russia). Low molecular weight chitosan (lactate, deacetylation degree >90%, average molecular mass 5 (4–6) kDa) was purchased from “Sigma-Aldrich” (St. Louis, MO, USA); sodium tripolyphosphate (STPP) was from Acros Organics (Fair lawn, NJ, USA); enalaprilat ((2S)-1-[(2S)-2-{[(1S)-1-carboxy-3-phenylpropyl]amino}propanoyl]pyrrolidine-2-carboxylic acid) was from U.S. Pharmacopeial Convention (Rockville, MD, USA); N-carbobenzoxy-L-phenylalanyl-L-histidyl-L-leucine (Cbz-Phe-His-Leu) was purchased from Bachem AG (Bubendorf, Switzerland); quercetin was from Himsnab-SPB (St. Petersburg, Russia); bovine ACE was purified as described in [39,40]. Ultrapure deionized water was used for all experiments.

All solutions used for the synthesis of the particles were preliminarily filtered using 0.45 μm syringe filters (Millipore, Germany), and the STTP solution was filtered using 0.2 μm filters (Millipore, Germany).

### 2.2. Enzyme Activity and Enalaprilat Concentration

The catalytic SOD1 activity was determined by quercetin assay [41]. ACE activity was determined by the rate of the hydrolysis of 0.2 mM Cbz-Phe-His-Leu at pH 7.5 [42].

Enalaprilat in different probes was determined by its ability to inhibit ACE activity. The curves of residual ACE activity on the dilution of analyzed solution were plotted; the dilution corresponding to 50% of ACE activity was determined and referred to enalaprilat concentration on preliminarily obtained calibration curve.

### 2.3. The Effect of Ultrasonic Treatment on SOD1 Activity

SOD1 solution (0.3 mg/mL) in 12.5 mM K_2_HPO_4_, 15.6 mM sodium citrate, and 25 mM NaCl, pH 8.8, was ultrasonicated at a power 200 W on the ultrasonic homogenizer (Bandelin Sonopuls, Germany) for 10 and 20 min. After that, the activity of SOD1 was determined as described above.

### 2.4. Optimization of SOD1-Loaded Calcium Phosphate Particles Preparation

SOD1-loaded CaP-particles were obtained using slightly modified co-synthesis method [17,19,43]. All the solutions necessary for the synthesis ((12.5 mM K_2_HPO_4_ containing 0.66 mg/mL (62.3 kU/mL) SOD1; 15.6–46.8 mM sodium citrate; 12.5 mM CaCl_2_)) were prepared and pre-filtered. Sodium citrate solution (1 volume) was added to K_2_HPO_4_ solution with SOD1 (5 volumes) and then the pH of the mixture was adjusted to 7.0, 7.5, or 8.8. Then, CaCl_2_ solution (5 volumes) was added simultaneously with the turning on the ultrasonic homogenizer for 20 min at 120, 160, or 200 W. The final concentration of SOD1 was 0.188 mg/mL. The synthesis was carried out without cooling, cooling with water at room temperature, and cooling with ice water. After that, the suspension was again filtered and stored at 4 °C.

### 2.5. Preparation of SOD1/Enalaprilat Co-Loaded Calcium Phosphate Particles

All the solutions necessary for the synthesis ((12.5 mM K_2_HPO_4_ containing 0.66 mg/mL (62.3 kU/mL) SOD1 and 2.75 mg/mL (8 mM) of enalaprilat; 15.6–46.8 mM sodium citrate; 12.5 mM CaCl_2_)) were prepared and pre-filtered. Sodium citrate solution (1 volume) was added to K_2_HPO_4_ solution with SOD1 and enalaprilat (5 volumes), and the pH was adjusted to 7.0. Then, CaCl_2_ solution (5 volumes) was added simultaneously with the turning on the ultrasonic homogenizer for 20 min at 200 W. The final concentration of SOD1 was 0.188 mg/mL and enalaprilat 0.76 mg/mL (2.2 mM). The synthesis was carried out by cooling with ice water. After that, the suspension was again filtered and stored at 4 °C.

Empty CaP-particles (without SOD1/enalaprilat) were prepared using a similar protocol.

### 2.6. Covering of CaP-Particles by Chitosan

Covering CaP-particles with chitosan was performed as described in [19]. Briefly, 1 volume of 1 mg/mL 5 kDa chitosan in deionized water, pH 3.9, was added dropwise to 2 volumes of CaP-particle suspension, pH 6.5, with constant vigorous stirring on a magnetic stirrer (400 rpm). Further, an aqueous solution of 1 mg/mL STPP (0.2 volume) was added dropwise to the reaction mixture. The system was left at constant stirring overnight at room temperature. The resulting suspension of hybrid chitosan-covered CaP-particles was filtered and stored at 4 °C. The percentage of chitosan in hybrid particles was estimated by the reaction of the interaction of free amino groups of chitosan with *ortho*-phthalaldehyde and N-acetyl-L-cysteine with the formation of a chromophore compound [44].

### 2.7. Dynamic Light Scattering (DLS) and Zeta Potential Measurements

CaP-particles and chitosan-covered CaP-particles were characterized by DLS using a Zetasizer Nano ZS instrument (Malvern Co., Ltd., UK) at 25 °C. The average hydrodynamic diameter and polydispersity index (PDI) of the particles were measured in 6 mM potassium chloride solution with 4 mW He-Ne laser of 633 nm wavelength using a backscatter angle of 173°. The given particle size data refer to scattering number distributions. The size distribution was processed using non-negative least squares analysis, NNLS (Malvern Co., Ltd., Dartford, UK). The ζ-potential was analyzed in water in specialized cuvettes with golden electrodes. The data were automatically processed using the Zetasizer v.7.03 software. Size and ζ-potential data were presented as the mean of a minimum of 3 values.

### 2.8. Morphology of CaP-Particles and Chitosan-Covered CaP-Particles

Uncovered CaP-particles and hybrid chitosan-covered CaP-particles, both without enalaprilat and SOD1, were studied by scanning electron microscopy (SEM) using dual beam, field emission scanning electron-ion microscope Scios, 1–30 kV (FEI, USA). Morphology and shape of the particles were determined on the samples pre-dialyzed against deionized water and freeze-dried on conductive double-sided carbon tape.

### 2.9. Fourier-Transform Infrared (FTIR) Spectroscopy

All the samples of empty CaP-particles, empty hybrid particles, SOD1/enalaprilat co-loaded CaP-particles, and SOD1/enalaprilat co-loaded hybrid particles were preliminarily filtrated and concentrated 20 times by centrifugation on 100 kDa membrane (Sartorius, Göttingen, Germany) to remove the excess of chitosan, SOD1, and enalaprilat. In addition, solutions of 5 kDa chitosan, SOD1, and enalaprilat were prepared at the same concentration as within the particles.

FTIR spectroscopy was carried out using a Tensor 27 instrument (Bruker, Bremen, Germany) equipped with a liquid nitrogen-cooled MCT detector, a thermostat (Huber, Offenburg, Germany), an attenuated total reflection cell (Bruker, Ettlingen, Germany) and a ZnSe single-reflection crystal. The spectra of the samples (40–50 μL) were recorded three times (70 scans each time) within a range of 3000–900 cm^−1^ at a resolution of 1 cm^−1^ at 22 °C. Dry air was pumped through the system by the air compressor (Jun-Air, Munich, Germany). The background (deionized water) was recorded in the same manner. The spectra were analyzed using the Opus 7.0 software.

### 2.10. Evaluation of SOD1 and Enalaprilat Loading into Hybrid Particles

In a standard protocol, a suspension of SOD1-containing hybrid particles was concentrated 10 times by centrifugation on 100 kDa membrane (Sartorius, Germany). The amount of unbound SOD1 in the filtrate was determined by its activity.

Similarly, a suspension of hybrid particles simultaneously containing SOD1 and enalaprilat was concentrated, and the amount of unbound SOD1 and enalaprilat was evaluated in the filtrate by SOD1 activity and enalaprilat’s ability to inhibit ACE.

### 2.11. In Vitro Release of SOD1 and Enalaprilat from Chitosan-Covered CaP-Particles

In a standard protocol, several portions of the suspension of SOD1-containing or SOD1- and enalaprilat-containing chitosan-covered CaP-particles (500 μL each) were concentrated on Sartorius filters (Germany) with a pore size of 100 kDa up to 50 μL to remove 90% of unbound SOD1 and enalaprilat. Then, the concentrate remaining above the filter was re-suspended by adding 450 μL of 150 mM NaCl solution and incubated for various time intervals (5, 15, 45, 90, 120 min) at room temperature. After the incubation, the suspensions were centrifuged at 5400 rpm for 5 min, and the SOD1 and enalaprilat were determined in the supernatants. All experiments were performed in triplicates.

### 2.12. Experiments In Vivo

In vivo studies were conducted using normotensive adult male Chinchilla rabbits weighing 2.0–2.5 kg. All experiments with live rabbits were carried out in strict accordance with the Association for Research in Vision and Ophthalmology (ARVO) statement for the Use of Animals in Ophthalmic and Vision Research. The protocol was approved by the Committee on the Ethics of Animal Experiments of the Helmholtz National Medical Research Center of Eye Diseases (Permit number 22/2).

#### 2.12.1. Retention of Free SOD1 and SOD1 within Hybrid Particles in the Lacrimal Fluid of Rabbits

A comparative assessment of the retention of free SOD1 and SOD1 within CaP-particles in the lacrimal fluid was carried out by determining SOD1 activity in tears after different time intervals after single instillations of corresponding solutions. Three independent series of experiments were carried out both for free SOD1 and SOD1-loaded CaP-particles covered with 5 kDa chitosan. In each experiment, animals obtained a single instillation of 50 µL SOD1 solution, pH 7.3, or 50 µL of a suspension of hybrid particles with the same SOD1 activity, pH 7.3, in both eyes.

In each series of experiments, 12 rabbits (24 eyes) were randomly divided into three groups of 3 animals each (6 eyes). The lacrimal fluid was collected before the experiment and at different time intervals after instillation with standard pieces of filter paper as described in [19]. So, the lacrimal fluid from 3 rabbits was collected after 10 min, from another 3 rabbits—after 30 min, and the lacrimal fluid from the rest 3 animals was collected after 1 h after instillation. Eluates from filter papers were prepared as in [19], and residual content of SOD1 in eluates was determined by its activity assessment.

#### 2.12.2. The Influence of Free Enalaprilat, Free SOD1, and SOD1 and Enalaprilat within Hybrid Particles on the Intraocular Pressure

A comparative assessment of the effects of water solutions of SOD1 and enalaprilat; SOD1 within hybrid particles, enalaprilat within hybrid particles, and co-loaded SOD1/enalaprilat within hybrid particles on IOP was carried out on normotensive rabbits. Two series of experiments were carried out; the instillations of the same solutions were conducted in both eyes. It is known that the processes in one eye can affect the fellow eye [45,46], which could be due to some sympathetic physiological response. This is why we did not use an approach where one eye of each animal is experimental while another eye serves as a control. Instead, each rabbit received instillations in both eyes.

In the first series, the animals were randomly divided into four groups: (1) experimental group 1 (4 rabbits, 8 eyes) received single instillations of 50 μL of the suspension of SOD1/enalaprilat co-loaded hybrid particles containing 0.188 mg/mL SOD1 and 0.76 mg/mL (2.2 mM) enalaprilat; (2) experimental group 2 (4 rabbits, 8 eyes) received single instillations of 50 μL of the suspension of SOD1-loaded hybrid particles with the same SOD1 concentration; (3) experimental group 3 (4 rabbits, 8 eyes) received single instillations of 50 μL of free SOD1 of the same concentration; (4) the control group (3 rabbits, 6 eyes) received single instillations of 50 μL of the suspension of empty hybrid particles.

In the second series of experiments, the animals were also divided into four groups: (1) experimental group 1 (4 rabbits, 8 eyes) received single instillations of 50 μL of the suspension of SOD1/enalaprilat co-loaded hybrid particles containing 0.188 mg/mL SOD1 and 0.76 mg/mL (2.2 mM) enalaprilat; (2) experimental group 2 (4 rabbits, 8 eyes) received single instillations of 50 μL of the suspension of enalaprilat-loaded hybrid particles with the same enalaprilat concentration; (3) experimental group 3 (4 rabbits, 8 eyes) received single instillations of 50 μL of free enalaprilat of the same concentration; (4) the control group (3 rabbits, 6 eyes) received single instillations of 50 μL of the suspension of empty hybrid particles.

IOP was measured before and after the instillations for 6 h at intervals of 1 h using a Tonovet automatic veterinary IOP monitor (Icare, Vantaa, Finland). IOP in normotensive rabbits was 15 ± 2 mm Hg. The effect of the preparations on IOP was evaluated as the difference between mean IOP in the control group and the mean IOP in experimental group.

### 2.13. Statistical Analysis

All experiments were conducted independently in duplicate or triplicate, the results were expressed as mean value ± standard deviation, SD. Statistica for Windows (version 10.0, Stat.Soft. Inc., Tulsa, OK, USA) was used for statistical analysis. Significance was analyzed by Mann–Whitney test with *p* ≤ 0.05 considered statistically significant and *p* ≤ 0.01 considered highly statistically significant.

## 3. Results and Discussion

### 3.1. Synthesis and Characterization of SOD1-Containing Hybrid Particles

First of all, we evaluated the thermal stability of SOD1 to be sure that the enzyme retained its activity at the synthesis conditions, as the temperature of the medium could rise to 60–70 °C under the action of ultrasound. SOD1 retained full enzymatic activity for an hour in 12.5 mM K_2_HPO_4_ containing 15.6 mM sodium citrate and 25 mM NaCl at temperatures up to 60 °C. At 70 °C, the SOD1 activity only slightly decreased during the first 20 min, but after an hour, SOD1 retained only half of its enzymatic activity.

In addition, 200 W ultrasonic treatment of the SOD1 solution for 20 min had no effect on SOD1 activity. Even though SOD1 was stable under ultrasound treatment, under the conditions of CaP-particles synthesis, we observed the formation of abundant stable foam, which was observed only in the presence of SOD1. So, we can conclude that the presence of CaP-nanoparticles in the suspension provoked foaming, which could proceed more intensively at the interface between the solution and solid particles with SOD1, as a protein, playing a role of a foam stabilizer [47]. Note that SOD1 activity did not decrease due to foam formation. However, as SOD1 was adsorbed on the foam, its concentration in the solution rapidly decreased, and therefore did not allow the enzyme to be loaded into newly formed CaP-nanoparticles. Moreover, the formation of the foam also significantly affected the process of particle formation, as the resulting CaP-particles were only about 30 nm compared to 80 nm CaP-particles formed without SOD1 in the medium [19], since surfactants can lead to a decrease in the resulting particles [48,49].

Reducing the sonication power to 160 W had no effect on foaming. A further decrease in power to the limiting 120 W required for the formation of particles slightly reduced the intensity of the foam, while the percentage of SOD1 inclusion into CaP-particles was still extremely low, only about 5%. In attempts to avoid foaming, we tried to prepare nanoparticles under cooling the synthesis vessel with ice water (4 °C). At these conditions, the mixture was heated to 20–23 °C after 20 min of 200 W ultrasonic treatment in contrast to 60–70 °C without cooling [19]. However, temperature decrease resulted in aggregation and precipitation of the particles; varying the pH of the reaction mixture did not improve the situation.

So, we changed tactics and increased the concentration of stabilizing agent sodium citrate in the reaction medium from 1.4 to 4.3 mM, which allowed us to obtain SOD1-containing CaP-nanoparticles at cooling. These particles were characterized by a mean hydrodynamic diameter of 80 nm, which is small enough for a drug delivery vehicle [50], but were rather unstable and did not withstand concentration and washing on filters. Thus, they were immediately covered with 5 kDa chitosan by ionotropic gelation with STPP as in [19] to increase their stability. The obtained empty uncoated and covered with 5 kDa chitosan particles retained their characteristics when kept in suspension at 4 °C for at least one month.

**Figure 1:**
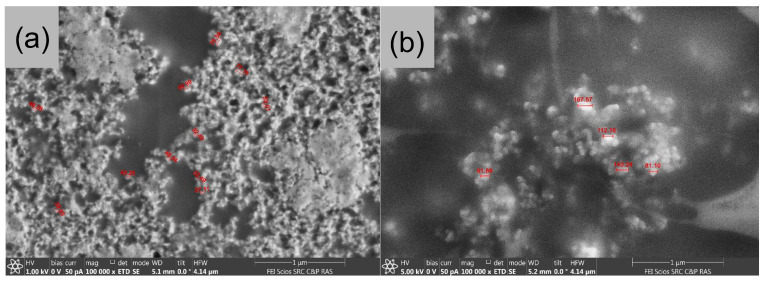
SEM-images of empty uncoated (**a**) and covered with 5 kDa chitosan (**b**) calcium phosphate (CaP) nanoparticles.

SEM study of uncovered CaP-nanoparticles obtained in the presence of 4.3 mM citrate and under cooling with ice water and then freeze-dried on conductive double-sided carbon tape showed many rounded particles with an average diameter of approximately 80 nm (Figure 1a) which is consistent with DLS data. Some smaller particles with a diameter of about 50 nm and some conglomerates formed during the drying were also observed. SEM images of CaP-nanoparticles obtained in the same conditions and further coated with 5 kDa chitosan were also characterized by a regular rounded shape and an average diameter of approximately 130 nm (Figure 1b), which coincided with DLS data.

The optimized synthesis conditions allowed us to effectively incorporate SOD1 into hybrid particles with a CaP-core and a 5 kDa chitosan coating. Under synthesis conditions, about 15% of chitosan of its total mass participated in the coating of CaP-nanoparticles. SOD1 did not lose its specific activity during synthesis and the percentage of SOD1 incorporation was high and reached 64% or 120 ± 5 μg SOD1 per 1 mg of hybrid particles. SOD1-containing particles were characterized by the particle size 150–190 nm measured by DLS and positive ζ-potential +22 mV indicating that the particles were effectively covered with chitosan (Table 1). Note that the chitosan “coat” cross-linked by STPP firmly adhered to CaP-nanoparticles, as the particles did not change their DLS characteristics after several washes on 30 kDa filters.

### 3.2. Co-Loading of SOD1 and Enalaprilat into Hybrid Particles

Preliminarily, we have shown that the presence of enalaprilat (up to 2.2 mM) did not affect SOD1 activity. Moreover, the presence of SOD1 (up to 0.3 mg/mL) did not affect the inhibitory activity of enalaprilat, which allows us to state that the simultaneous incorporation of these drugs into CaP-nanoparticles would not impede their activity and putative therapeutic action.

The incorporation of two preparations of different natures into CaP-nanoparticles was carried out in situ by the method of co-precipitation during ultrasonic treatment under the conditions of the introduction of SOD1 alone. Immediately after the synthesis, the particles were coated with 5 kDa chitosan to increase their stability. As a result, we obtained a suspension of hybrid particles simultaneously loaded with SOD1 and enalaprilat.

The hybrid particles co-loaded with SOD1/enalaprilat were characterized by a slightly reduced mean hydrodynamic diameter of about 140 nm compared to the particles containing SOD1 alone at 170 nm (Table 1). The co-loading of SOD1 and enalaprilat into particles resulted in an approximate twofold reduction in SOD1 content (Table 1), while the efficiency of enalaprilat incorporation only slightly decreased. The inclusion of enalaprilat into the particles from the mixture of SOD1 and enalaprilat is more preferable due to its much smaller size together with the interaction of negatively charged carboxyl groups of enalaprilat with both calcium ions in inorganic core and protonated amino groups of chitosan cover. Thus, the SOD1 content was 56 ± 5 μg and the enalaprilat content was 430 ± 50 μg per 1 mg of the co-loaded hybrid particles.

It should be noted that, while uncoated CaP-particles had negative ζ-potential −18 mV, chitosan-covered CaP-particles were characterized by a positive charge, indicating that the chitosan coating was efficiently produced. The value of ζ-potential was much higher in the case of SOD1-loaded and SOD1/enalaprilat-loaded particles (Table 1). Such a sufficiently large positive charge is beneficial as it is necessary for the particle’s affinity to the eye surface [51].

These SOD1-loaded and co-loaded SOD1/enalaprilat hybrid particles could be stored as a suspension for at least a month at 4 °C with no change in their characteristics, as well as previously studied enalaprilat-loaded ones [19]. SOD1 inclusion did not affect the stability of the particles.

### 3.3. Fourier-Transform Infrared (FTIR) Spectroscopy

**Figure 2:**
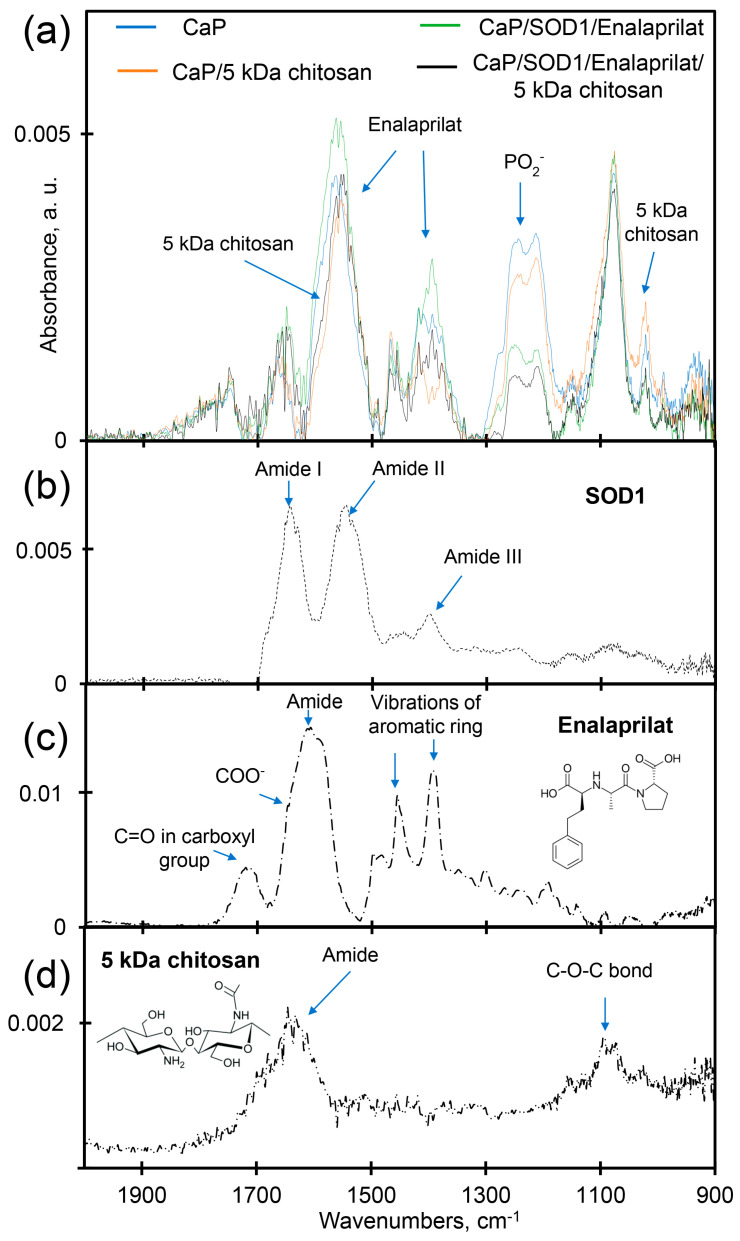
ATR-FTIR spectra of (**a**) CaP-particles (blue), empty hybrid particles (orange), SOD1/enalaprilat co-loaded CaP-particles (green), SOD1/enalaprilat co-loaded hybrid particles (black); (**b**) SOD1, (**c**) enalaprilat, (**d**) 5 kDa chitosan.

Attenuated total reflectance (ATR)-FTIR spectrum of the co-loaded SOD1/enalaprilat CaP-particles covered with 5 kDa chitosan (Figure 2a) demonstrate the presence of all compounds—SOD1, enalaprilat and chitosan—within the particles. The incorporation of SOD1 and enalaprilat into CaP-particles resulted in a significant increase in the intensity in the regions of 1700–1600 cm^−1^ and 1600–1500 cm^−1^, which can be attributed to the amide I and amide II of SOD1 [52,53] and vibrations of the aromatic structure of enalaprilat (Figure 2b,c). In addition, the inclusion of drugs in hybrid and CaP-particles was also evidenced by the intensity increase in the 1415–1375 cm^−1^ region, which can be attributed to the SOD1 and enalaprilat aromatic ring vibrations.

The coating of empty CaP-particles and co-loaded SOD1/enalaprilat CaP-particles resulted in a decrease in intensity (quenching) in the 1600–1500 cm^−1^ region and the shift of the band at 1620–1700 cm^−1^ to low-frequency region due to the interaction of the particles with polysaccharide chitosan. The interaction of CaP-particles with the chitosan “coat” is also clearly indicated by the dramatic intensity decrease in the 1260–1220 cm^−1^ region after particle covering. This is the main analytically significant band in CaP-particles—absorption band of the valent oscillations of the phosphate group (1220–1260 cm^−1^) [54], the main site of CaP-particles interaction with cationic groups of chitosan and the protein, SOD1 [53,54,55]. At the same time, the intensity of the absorption peak observed for CaP-particles at 950 cm^−1^ (corresponding to C⎯N bonds vibrations in chitosan [52]) increased as a result of chitosan covering, whereas the incorporation of SOD and enalaprilat into the particles resulted in a decrease in the intensity.

Thus, FTIR-spectra clearly demonstrated that chitosan is a part of hybrid particles, while SOD1 and enalaprilat are present within both uncovered CaP-particles and CaP-particles covered with chitosan.

### 3.4. SOD1 and Enalaprilat Release from Hybrid Particles

The kinetics of drug release from the carrier is an important parameter for its further use in vivo, since it can affect the pharmacokinetics, bioavailability, and therapeutic efficacy of the drug. The kinetics of SOD1 and enalaprilat release from co-loaded hybrid particles was studied in 0.15 M NaCl solution in order to simulate ionic strength typical for most biological fluids. For this purpose, the particles were separated from the excess of free SOD1 and enalaprilat by filtration and placed into a fresh solution of NaCl. The SOD1 release into solution was monitored by its activity and the enalaprilat release was monitored by its ability to inhibit control ACE.

**Figure 3:**
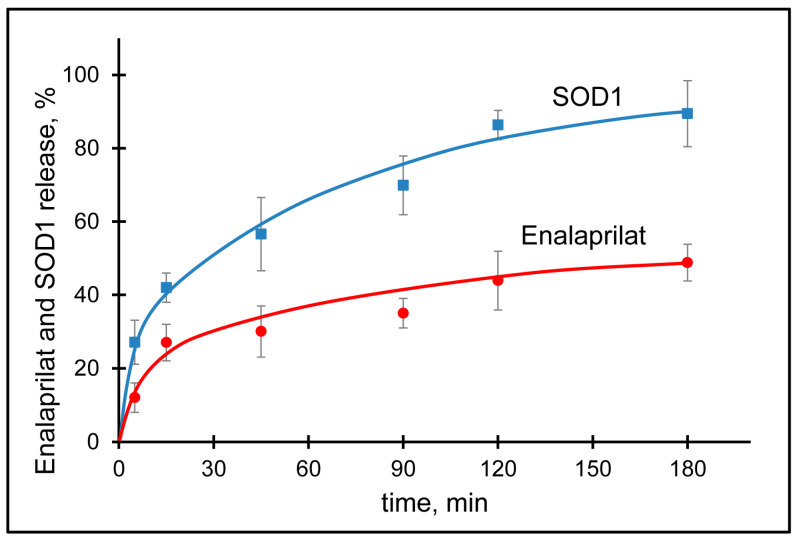
Elution of enalaprilat (red) and SOD1 (blue) from CaP-nanoparticles covered with 5 kDa chitosan in 0.15 M NaCl, pH 7.5.

The SOD1 release profile showed a burst (fast release of about 40%) within the first 15 min followed by a sustained release reaching the plateau (more than 80% release) by the third hour (Figure 3).

Meanwhile, the release of enalaprilat from the particles is a prerequisite for the use of enalaprilat-containing particles in vivo, since enalaprilat should be able to reach its target, ACE, in the tissues of the eye. The enalaprilat release profile was rather smooth with a 25% burst within the first 15 min followed by a sustained release reaching the plateau (almost 50%) by the second hour at about 44% (Figure 3). The release profile of enalaprilat from co-loaded particles appeared to be very similar to that for enalaprilat incorporated within hybrid particles without SOD1 [19]. The complete release of enalaprilat from the particles occurred after incubation of the particles in a fresh portion of the eluate.

To find out the possible mechanism of SOD1 and enalaprilat release from CaP-particles, the data were fitted on different mathematical models: Korsmeyer–Peppas, First order, Higuchi, and Hixson–Crowell [56]. The most suitable model was selected using the correlation coefficient (R^2^). We found that the release of both SOD1 and enalaprilat from the hybrid particles best fitted the Korsmeier–Peppas model. That is, we observed a diffusion-controlled release of substances from the developed carrier, and the parameter “n” in this model serves as an indicator of the degree of a diffusional release. The value of “n” for the SOD1 and enalaprilat release from the hybrid particles was 0.32 and 0.39, respectively, indicating a diffusion-controlled release of both SOD1 and enalaprilat (corresponds to the Fickian diffusion).

### 3.5. Retention of Free SOD1 and SOD1 within Hybrid Particles in the Lacrimal Fluid

Another important property of the drug carrier is its ability to increase the retention time of the substance in the lacrimal fluid and, as a result, the contact of the substance with the cornea. Previously, we have shown that the inclusion of enalaprilat in hybrid CaP-nanoparticles covered with 5 kDa chitosan can extend its retention time in the lacrimal fluid and some amount of enalaprilat was still detected an hour after instillation [19].

**Figure 4:**
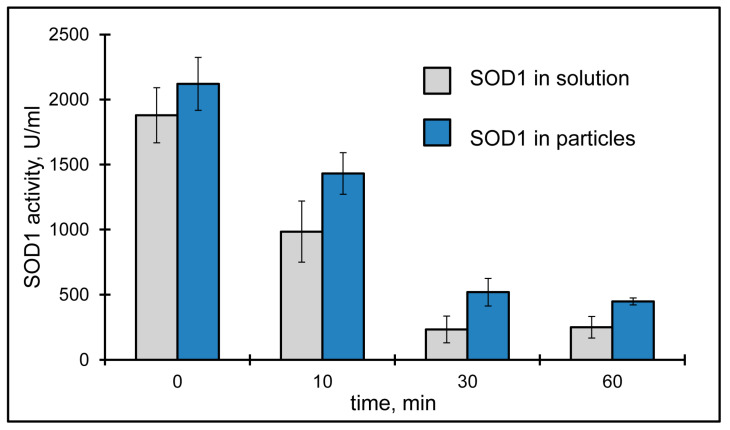
SOD1 activity in lacrimal fluid after single instillation of SOD1-containing hybrid particles (blue) and SOD1 solution (grey).

The initial removal of SOD1 from the lacrimal fluid appeared to be very fast. It is noteworthy that the basal tear volume (usually less than 10 μL [57]) is significantly less than the drop volume (50 μL); therefore, the excess of the instilled drug immediately outflows from the eye surface. SOD1 activity instilled as a suspension of hybrid particles appeared to be higher 10 min after the instillation than SOD1 activity instilled as SOD1 solution (Figure 4). Moreover, we could find some SOD1 activity in the lacrimal fluid even half an hour after the instillation of the suspension of SOD1-containing hybrid particles, while free SOD1 seemed to be totally washed out by this time (Figure 4). So, SOD1 inclusion into chitosan-covered CaP-nanoparticles helped to increase SOD1 “lifetime” in the lacrimal fluid. After an hour, SOD1 activity returned to the level of that for endogenous SOD1.

### 3.6. Effects of Topical Instillations of Enalaprilat and SOD1 in Solutions and within Hybrid Particles on the Intraocular Pressure

In each series of experiments, the animals were randomly divided into four groups: (1) first experimental group received single instillations of the suspension of hybrid particles containing both SOD1 and enalaprilat into both eyes of the animal; (2) second experimental group received single instillation of hybrid particles of only one component—SOD1 or enalaprilat; (3) third experimental group received instillations of the solution of either SOD1 or enalaprilat; and (4) control group received single instillations of the suspension of empty hybrid particles.

Note that we did not remove an excess of SOD1 and enalaprilat after synthesis of the particles; that is, as an example, SOD1 activity in the suspension of SOD1-containing particles corresponded to the sum of the activity of SOD1 within the particles and SOD1 activity in the surrounding solution. Again, the content of enalaprilat in the suspension of the particles is the sum of enalaprilat content in the particles and the content of enalaprilat in the surrounding solution.

As we previously noted [19], empty hybrid particles neither affected IOP nor irritated the eye.

**Figure 5:**
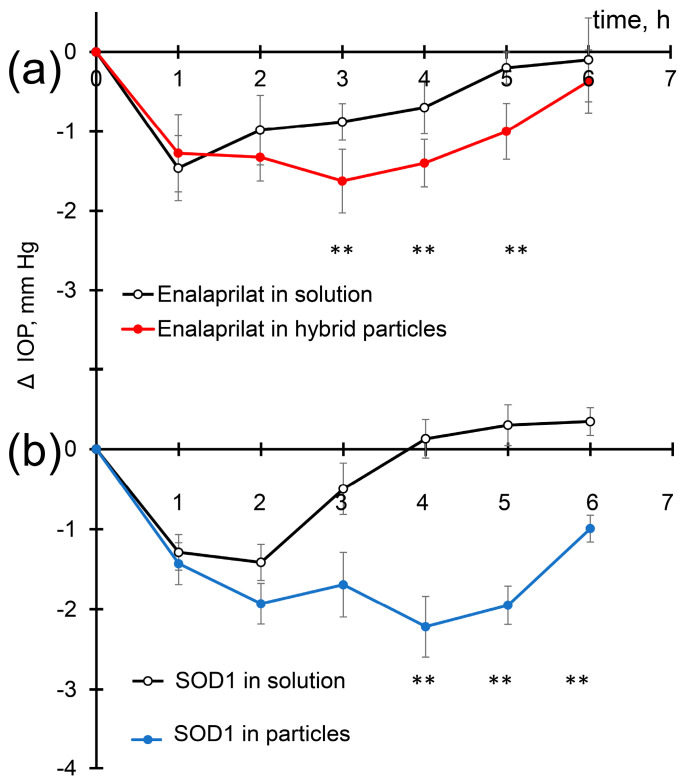
Intraocular pressure (IOP) decrease in normotensive rabbits after single instillations of (**a**) enalaprilat-loaded particles and enalaprilat in aqueous solution; (**b**) SOD1-loaded hybrid particles and SOD1 in aqueous solution. ** *p* < 0.01.

Enalaprilat in solution and within hybrid particles caused a significant decrease in IOP (Figure 5a) in accordance with previous observations [19]. One hour after the instillation of enalaprilat solution, the decrease in IOP was approximately 1.4 mm Hg, similar to the decrease caused by enalaprilat loaded into the particles. Two hours after the instillation of enalaprilat in solution, IOP values began to rise and reached the initial values after five hours. Two hours after the instillation of enalaprilat within hybrid particles, however, IOP further decreased and reached its minimum at 3 h; IOP decrease being equal to −1.6 mm Hg. Even after 6 h, we observed decreased IOP (Figure 5a). Thus, enalaprilat in the form of hybrid particles led to a more pronounced, prolonged, and statistically significant decrease in IOP.

Similarly, the use of SOD1 both in the free form and as a part of hybrid particles led to a significant decrease in IOP about 1.4 mm Hg one hour after instillation (Figure 5b). The effect of SOD on IOP is even more pronounced than enalaprilat. This IOP decrease caused by SOD1 in solution was similar to that caused by enalaprilat in solution at chosen SOD1 and enalaprilat concentrations. Two hours after the instillation of SOD1 in solution, IOP remained at the same level, but after 3 h, the instillation almost returned to the initial value. Meanwhile, IOP of the eyes instilled with SOD1 loaded into the particles continued to decrease and reached a minimum (−2.2 mm Hg) at 4 h. We should especially note the remarkable prolongation of SOD1 action within hybrid particles (Figure 5b). The IOP in rabbits treated with SOD1-loaded particles remained reduced even 6 h after instillation (Figure 5b).

**Figure 6:**
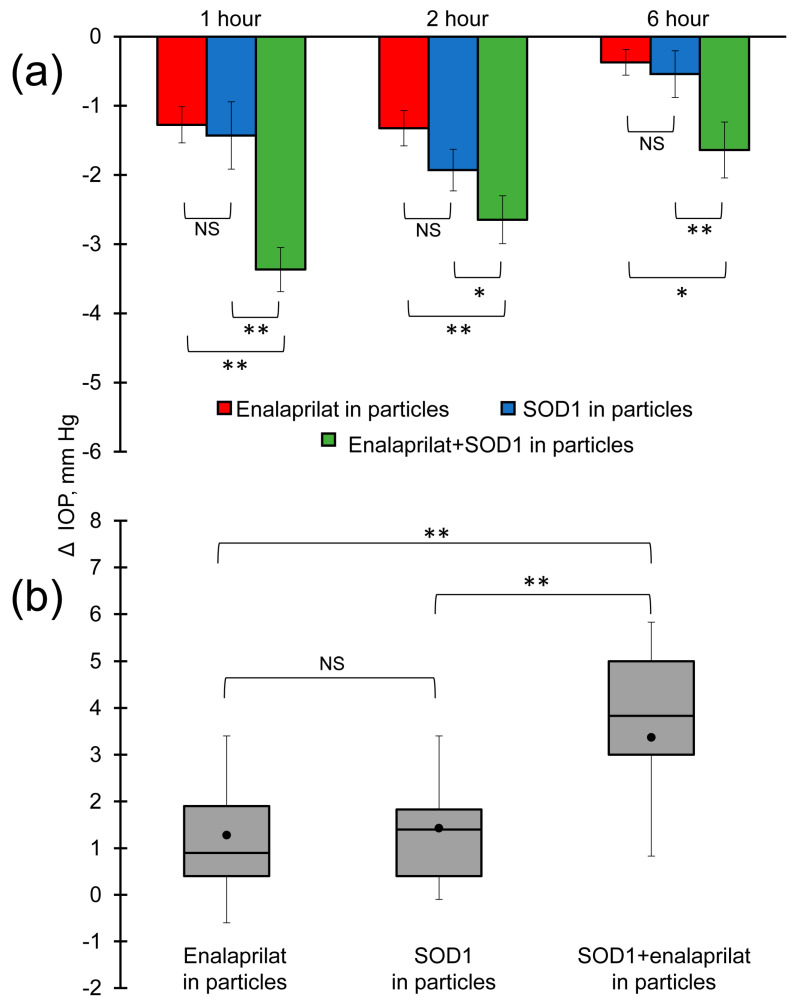
(**a**) IOP decrease in normotensive rabbits after single instillation of enalaprilat-loaded hybrid particles (red), SOD1-loaded hybrid particles (blue) and SOD1/enalaprilat co-loaded hybrid particles (green). (**b**) Comparison of IOP decrease in normotensive rabbits an hour after single instillation of enalaprilat-loaded hybrid particles, SOD1-loaded hybrid particles, and SOD1/enalaprilat co-loaded hybrid particles. The mean is marked with a dot. Value interval 25–75% is indicated by the grey area. * *p*< 0.05, ** *p*< 0.01.

The most important observation was that, at chosen concentrations, instillations of SOD1 within hybrid particles and enalaprilat within hybrid particles led to a similar drop in IOP of about 1.4 mm Hg after an hour past instillation. However, the instillation of SOD1/enalaprilat co-loaded hybrid particles resulted in a significant IOP decrease by almost 3.5 mm Hg after an hour past instillation (Figure 6a,b). If the effects of SOD1-loaded particles and enalaprilat-loaded particles were summarized, the sum would be equal to 2.8 mm Hg. Thus, in the first hour after instillation, co-loaded SOD1 and enalaprilat within hybrid particles caused some synergistic effects, which appear to be due to the different physiological mechanisms of action of SOD1 and enalaprilat. While enalaprilat needs to be released from the particles to reach its target, ACE, in the eye tissues, SOD1 can act both when released from the particles and while remaining in them, because low molecular weight reactive oxygen species can penetrate the particles and reach SOD1 within them. We found that, at the first hour, it was SOD1 that made the main contribution to the decrease in IOP. At the second hour, the synergy disappeared, but the effect of SOD1/enalaprilat co-loaded hybrid particles on IOP was still remarkable and statistically significant (Figure 6b). Moreover, a statistically more pronounced effect of SOD1/enalaprilat within hybrid particles on IOP persisted even 6 h after instillation.

## 4. Conclusions

In this study, we optimized the conditions of CaP-core synthesis, which made it possible to co-load two components, antioxidant enzyme, SOD1, and low molecular weight ACE inhibitor, enalaprilat, into the nanoparticles without losing SOD1 activity. Further covering of the particles with 5 kDa chitosan made it possible to obtain stable and rather small, about 140 nm, hybrid particles with a narrow size distribution. Such particles had a high loading capacity of enalaprilat (56%), and at the same time were capable of including a sufficiently large amount of SOD1 (30%).

An enalaprilat and SOD1 release from CaP-nanoparticles covered with 5 kDa chitosan in physiologically relevant conditions proceeds for tens of minutes, providing both the retention of both drugs within the vehicle, necessary for effective drug delivery into the eye, and the release, necessary at least for enalaprilat to find its target, ACE, in the ACE tissues.

The suggested formulation effectively, and for a long time, decreases intraocular pressure, which can allow a decrease in the dose and frequency of drug administration and, therefore, reduce side effects and increase patient compliance. Moreover, this formulation combines hypotensive action with anti-ischemic and antioxidant properties, which is important for neuroprotection and the saving of vision.

## Figures and Tables

**Table 1:** Characteristics of obtained nanoparticles.

Cover	Loaded Substance	d, nm	PDI	ζ, mV	SOD1 Inclusion, %	Enalaprilat Inclusion, %
-	-	80 ± 20	0.36	−18 ± 4	-	-
5 kDa chitosan	-	155 ± 25	0.16	+16 ± 2	-	-
5 kDa chitosan	SOD1	170 ± 20	0.47	+22 ± 1	64 ± 3	-
5 kDa chitosan	Enalaprilat	180 ± 30	0.25	+7 ± 3	-	66 ± 5
5 kDa chitosan	SOD1/enalaprilat	140 ± 20	0.43	+20 ± 1	30 ± 2	56 ± 6

## Data Availability

Not applicable.
